# Author’s Reply to Professionalism—The Role of Quality Improvement

**DOI:** 10.5041/RMMJ.10220

**Published:** 2015-07-30

**Authors:** Paul S. Mueller

**Affiliations:** Consultant, Division of General Internal Medicine, Mayo Clinic, Rochester, MN, USA; Professor of Medicine and Professor of Biomedical Ethics at the Mayo Clinic College of Medicine, Rochester, MN, USA; Associate Editor of NEJM Journal Watch General Medicine

## TO THE EDITOR

I appreciate Dr Walsh’s feedback regarding my recent article, “Teaching and Assessing Professionalism in Medical Learners and Practicing Physicians.”^1^ I agree with Dr Walsh that quality improvement is a topic of importance within the professionalism domain.

Competent and trustworthy physicians who act for the benefit of patients naturally embrace quality improvement. As mentioned in my article, the Accreditation Council for Graduate Medical Education lists “accountability,” “commitment to excellence,” and “responsiveness” in its definition of *professionalism*. These attributes and behaviors are necessary for quality improvement. The Physician Charter on Medical Professionalism explicitly lists “commitment to improving quality of care” as one of ten “professional responsibilities.”

Nonetheless, I agree with Dr Walsh that I could have “made more of” the linkage between professionalism and quality improvement. As noted by Dr Walsh, emphasizing quality improvement has several advantages: it provides tangible and practical projects for physicians to improve themselves and the outcomes of their patients; it encourages physicians to support and participate in team activities; and it can be used as a metric for physician performance (e.g. quality of care delivered). In other words, quality improvement is a means by which professionalism is put into action.

Notably, at Mayo Clinic, quality improvement is taught at all levels of learning. For example, our internal medicine residents are required to undergo quality improvement training and participate in a quality improvement project by the end of their training.^2^ Our faculty members are required to complete basic quality improvement training; leaders are required to complete more advanced training. In fact, advanced quality improvement projects completed by Mayo Clinic faculty members satisfy maintenance of certification requirements of the American Board of Family Medicine, the American Board of Internal Medicine, and the American Board of Pediatrics.^3^

Dr Walsh correctly asserts, “… anything that can help all professionals improve their practice should also be able to help them with their professionalism.” The converse is also true. Anything that enhances professionalism should also facilitate quality improvement. However, I am less certain about viewing professionalism through a “prism of quality improvement.” Rather, all that we teach and assess in medical learners and practicing physicians—clinical competence, communication skills, sound ethics, and the aforementioned attributes of professionalism—and all that we do in medicine, including quality improvement, should be viewed through a “prism of professionalism” in order to determine whether we are meeting our professional responsibilities to patients, learners, colleagues, and society.

## References

[1] Mueller PS (2015). Teaching and assessing professionalism in medical learners and practicing physicians. Rambam Maimonides Med J.

[2] Leenstra JL, Beckman TJ, Reed DA (2007). Validation of a method for assessing resident physicians’ quality improvement proposals. J Gen Intern Med.

[3] Graves K (2010). Mayo Clinic recognized by 3 certifying boards for quality improvement. Ann Fam Med.

